# Food combination questionnaire for Japanese: relative validity regarding food and nutrient intake and overall diet quality against the 4-day weighed dietary record

**DOI:** 10.1017/jns.2023.7

**Published:** 2023-02-15

**Authors:** Kentaro Murakami, Nana Shinozaki, M. Barbara E. Livingstone, Nana Kimoto, Shizuko Masayasu, Satoshi Sasaki

**Affiliations:** 1 Department of Social and Preventive Epidemiology, School of Public Health, University of Tokyo, Tokyo 113-0033, Japan; 2 Nutrition Innovation Centre for Food and Health (NICHE), School of Biomedical Sciences, Ulster University, Coleraine BT52 1SA, UK; 3 Ikurien-Naka, Ibaraki 311-0105, Japan

**Keywords:** Dietary assessment, Japan, Overall diet, Rapid assessment, Validity, DR, dietary record, FCQ, food combination questionnaire, HEI-2015, Healthy Eating Index-2015, NRF9.3, Nutrient-Rich Food Index 9.3

## Abstract

The aim of this cross-sectional study was to examine the relative validity of food and nutrient intakes and overall diet quality scores derived using a newly developed dietary assessment questionnaire (food combination questionnaire, FCQ). Dietary data were collected from 222 Japanese adults (111 for each sex) aged 30–76 years using the online FCQ and then the 4-non-consective-day weighed dietary record (DR). The median of Spearman correlation coefficients for sixteen food groups was 0⋅32 among women and 0⋅38 among men. The median of Pearson correlation coefficients for forty-six nutrients was 0⋅34 among women and 0⋅31 among men. The Pearson correlation coefficient between the total scores of Healthy Eating Index-2015 (HEI-2015) derived from the DR and FCQ was 0⋅37 among women and 0⋅39 among men. The corresponding value for the Nutrient-Rich Food Index 9.3 (NRF9.3) total scores was 0⋅39 among women and 0⋅46 among men. Bland–Altman plots for these diet quality scores showed poor agreement at the individual level, although mean difference was small for the HEI-2015 (but not NRF9.3). Similar results were obtained using the paper version of FCQ, which was answered after conducting the DR, except for somewhat high Pearson correlation coefficients for the total scores of HEI-2015 (0⋅50 among both women and men) and NRF9.3 (0⋅37 among women and 0⋅53 among men). In conclusion, this analysis may lend support to the possible use of the FCQ as a rapid dietary assessment tool in large-scale epidemiologic studies in Japan, but further refinement of this tool should be pursued.

## Introduction

The improvement of diet quality is currently a global priority, since it has been widely acknowledged that dietary intake is a major determinant of morbidity and premature death^(1)^. An accurate assessment of habitual dietary intake is essential for investigating the dietary aetiology of chronic diseases and for promoting favourable changes in dietary behaviours^(2)^. The two dietary assessment methods, namely the dietary record (DR) and 24-h dietary recall, are acknowledged as the most widely used methods for capturing intakes of a wide variety of foods and nutrients^(3)^. For the assessment of habitual intake at the individual level, however, DR and 24-h dietary recall are usually conducted over multiple days, which is not always feasible due to expense and added participant burden^(3)^ despite the advancement of technology in recent years^(4)^. As a result, dietary assessment questionnaires are a most commonly used tool in large-scale epidemiologic and intervention studies to capture dietary intake^(5,6)^. Unlike the DR and 24-h dietary recall, dietary assessment questionnaires can capture long-term dietary intake in a single administration and are less cumbersome to complete^(7)^. However, it should be noted that dietary assessment questionnaires do not collect information on actual dietary intake itself but ultimately measure only the memory and perception of usual diet^(8)^. Consequently, a fit-for-purpose dietary assessment questionnaire requires a careful development and validation evaluation process.

We have recently developed a food combination questionnaire (FCQ) as a tool for assessing dietary habits in the Japanese population^(9)^. The FCQ has several unique and novel characteristics. First, the FCQ is data-driven, meaning that the development of the questionnaire structure, selection of food items and dietary intake calculation algorithms and database were informed by detailed dietary information derived from the 16-day weighed DR obtained from 242 Japanese adults^(9,10)^. Second, the FCQ assesses dietary intake for each meal type (i.e. breakfast, lunch, dinner and snacks) separately. This is mainly due to our observations in the Japanese population, in which the selection, amount and combination of foods consumed are markedly different between meal types^(11–13)^. Third, the FCQ predominantly focuses on the combination of foods which are consumed; thus, the primary aim of the FCQ is to collect information which is sufficient to distinguish food combinations for each meal type as in the most efficient way as possible. Finally, while the FCQ provides data on food combinations, for which standardised analytic procedures and techniques remain established^(2)^, the FCQ also provides data on a set of intake estimates of common food groups and nutrients, because the standard food composition database is incorporated in the data processing system of FCQ. While a previous study examined the validity of the FCQ against a well-established dietary assessment questionnaire^(9)^, a rigorous validation investigation against a more detailed dietary assessment method has not yet been conducted. Here, we present the relative validity of the FCQ with regard to food group and nutrient intakes and overall diet quality scores against the 4-day weighed DR.

## Methods

### Study procedure and participants

The survey procedure and participants have been described in detail elsewhere^(14)^, and only a brief description is provided here. We determined the sample size primarily based on the recommendation made by Cade *et al.* (i.e., more than 100 for each analysis)^(3)^, without any formal calculation. Between August and October 2021, the survey was conducted in fourteen (of the forty-seven) prefectures by sixty-six research dietitians with DR collection experience^(15,16)^. The fourteen prefectures were selected based on their geographic diversity (from Hokkaido to Kyushu) and the availability of experienced dietitians throughout the Japanese archipelago. In each prefecture, eight apparently healthy women (two from each of four age categories: 30–39, 40–49, 50–59 and 60–69 years) and their live-in husbands were recruited using a snowball sampling procedure, resulting in 112 invitations for each sex (not considering the age of the men). Although dietary data from cohabiting couples may reduce gender differences in dietary intake, we chose *a priori* to separate all analyses into women and men, so we do not consider this issue problematic in this study. Excluded from the study were single individuals, dietitians, individuals living with a dietitian, those who had received dietary counselling from a doctor or dietitian, those taking insulin treatment for diabetes, those undergoing dialysis treatment, those without sufficient internet access, those who had difficulty answering the web-based questionnaires, and pregnant or lactating women. In total, 111 women aged 30–69 years and 111 men aged 30–76 years completed the study. Due to the use of snowball sampling procedure, the number of individuals approached for this study and the number of individuals excluded from this study were not formally recorded.

First, participants were asked to complete the web FCQ created by Google Forms, with non-response not permitted. Seven to ten days after completion of the web FCQ, each participant completed the weighed DR for 4-non-consective days within 2 weeks. Finally, at intervals of at least 1 d, they were asked to complete the paper version of the FCQ, a A4 4-page questionnaire.

The study was conducted in accordance with the guidelines of the Declaration of Helsinki, and all procedures involving humans were approved by the Ethics Committee of the University of Tokyo Faculty of Medicine (protocol code: 2020326NI; date of approval: 29 January 2021). Written informed consent was obtained from all participants.

### Food combination questionnaire

Details of the development process of the FCQ and its structure and content have been published elsewhere^(9)^. Briefly, the FCQ was developed based on a food combination database which was based on the 16-day weighed DR data collected from 242 Japanese adults aged 31–81 years^(10)^. This DR dataset included 3788 breakfasts, 3823 lunches, 3856 dinners and 3267 snacks. For each meal type, we applied the frequent item sets data-mining methods^(17)^ for identifying the most commonly consumed combinations of seventeen selected food groups, obtaining eighty generic meals (or meal codes): twenty-three for breakfast, twenty-two for lunch, twenty-four for dinner and twelve for snacks^(9)^.

**Fig. 1.**
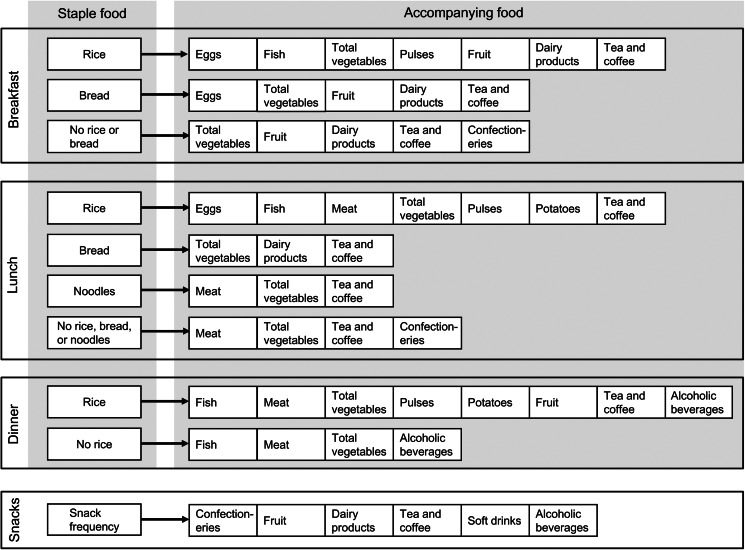
Structure of the food combination questionnaire (FCQ). In the FCQ, consumption frequency of each staple food in each meal type was enquired about in terms of the number of days with consumption per week during the preceding month; for snacks, consumption frequency was similarly enquired about without specifying any staple foods. For accompanying foods for each staple food, relative consumption frequency was enquired about, namely how often the food was consumed with the staple food, with the possible answers of ‘always’, ‘sometimes’ and ‘never’. For snacks, relative consumption frequency of selected foods was similarly enquired about. The food group ‘fish’ includes shellfish; the food group ‘pulses’ includes nuts.

The ultimate purpose of the FCQ is to collect information which is sufficient to distinguish food combinations using as few questions as possible^(9)^. A scrutiny of the food combination database showed that almost all generic meals (meal codes) except for snacks included one staple foods (i.e. rice, bread or noodles). Thus, we decided that in the FCQ, questions on staple foods are followed by questions on accompanying foods for each meal type (Fig. 1). Informed by the generic meals (meal codes) identified in the food combination database, staple foods included in the FCQ were rice and bread for breakfast; rice, bread and noodles for lunch; rice for dinner; and no staple food for snacks. For each staple food for each meal type, accompanying foods were then determined as food groups which had contributed to the determination of generic meals (meal codes).

In the FCQ (Fig. 1), we asked about consumption frequency (during the preceding month) as the number of days the food was consumed per week for each staple food for each meal type; for snacks, consumption frequency was asked about in a similar way without specifying any staple foods. For accompanying foods, we asked about relative consumption frequency, namely how often the food was consumed with the staple food, with possible answers of ‘always’, ‘sometimes’ and ‘never’. For snacks, relative consumption frequency of selected foods was similarly asked. According to a pretest conducted among nineteen individuals, the completion of the FCQ (paper version) generally took 5 min^(9)^.

The two delivery modes of FCQ used in this study (web FCQ and paper FCQ) are identical in terms of content. All responses to the web FCQ, which were automatically assigned in spreadsheet format, were downloaded from Google Drive. For the paper FCQ, responses to all questions were checked by the research dietitians and staff at the study centre. If any responses were missing, the participants were asked to answer the questions again in person or by phone. All answers in the paper FCQ were manually entered into a spreadsheet in duplicate, and any disagreement was checked and corrected.

On the basis of a series of *ad hoc* computer algorithms in the FCQ and the food combination database^(9,10)^, estimated intakes of food groups were calculated. As the food combination database was developed based on the 2015 version of the Standard Tables of Food Composition in Japan^(18)^, estimated intakes of selected nutrients were then calculated. Component scores needed for the calculation of the Healthy Eating Index-2015 (HEI-2015) were calculated using the Japanese version^(19)^ of the US Food Patterns Equivalents Database^(20)^. The calculation was done for each meal type, and the overall intake was calculated as the sum of the intake of each meal type.

### Weighed dietary record

The 4-non-consecutive-day weighed DR was selected as the reference method in this validation study. Each recording period consisted of three weekdays (Monday–Friday, except for national holidays) and one weekend day (Saturday, Sunday or national holidays). For each couple, a recording day was allocated within two weeks by research dietitians. Each couple was provided with recording sheets and a digital scale (KS-274, Dretec, Japan; ±2 g precision for 0–500 g and ±3 g precision for 500–2000 g). After receiving written and verbal instructions from the assigned research dietitian, as well as an example of a completed diary sheet, each participant was requested to document and weigh all consumed foods and drinks, both inside and outside of their homes, on each recording day. On certain occasions when weighing was inconvenient to carry out (e.g. dining out), they were instructed to document as much information as possible, including the brand name of the food and the consumed portion size (based on typical household measures), as well as the details of the leftovers.

The recording sheets used in each survey day were submitted directly to the research dietitian after the survey was completed, who then reviewed the forms and, whenever necessary, sought additional information or modified the record via phone or in-person interview. All collected records were then reviewed by the research dietitians and trained staff at the study centre. In accordance with a standardised procedure, the portion sizes estimated using household measures were converted into weights, and the individual food items were coded based on the 2015 version of the Standard Tables of Food Composition in Japan^(18)^. A total of 1297 food codes were used in the DR. Estimated intakes of food groups, energy, selected nutrients and component scores needed for the calculation of HEI-2015 were calculated using the 2015 version of the Standard Tables of Food Composition in Japan^(18)^ and the 2011–2012 Food Patterns Equivalents Database^(19,20)^. For all dietary variables, the mean daily values within the 4-day period were used for each individual.

### Calculation of diet quality scores

As measures of overall diet quality, we used the HEI-2015^(21–23)^ and Nutrient-Rich Food Index 9.3 (NRF9.3)^(24–27)^. As described elsewhere^(21–23)^, HEI-2015 is a composite measure of compliance with the 2015–2020 Dietary Guidelines for Americans^(28)^. The HEI-2015 is a 100-point scale, with a higher score indicating a better quality of diet. The HEI-2015 consists of nine adequacy components, namely, total fruits (maximum score: 5), whole fruits (5), total vegetables (5), greens and beans (5), whole grains (10), dairy products (10), total protein foods (5), seafood and plant proteins (5), and fatty acids as the ratio of the sum of polyunsaturated fatty acids and monounsaturated fatty acids to saturated fatty acids (10), and four moderation components, namely, refined grains (10), sodium (10), added sugars (10) and saturated fats (10). We calculated the HEI-2015 component and total scores based on energy-adjusted values of overall dietary intake, namely, amount per 4184 kJ (1000 kcal) of energy or percentage of energy, except for the fatty acids component^(19)^.

As described in detail elsewhere^(24–27)^, the NRF9.3 is a composite measure of the nutrient density of the diet, calculated as the sum of the percentage of reference daily values for nine qualifying nutrients, namely, protein, dietary fibre, vitamin A, vitamin C, vitamin D, calcium, iron, potassium and magnesium, minus the sum of the percentage of reference daily values for three disqualifying nutrients, namely, added sugars, saturated fats and sodium. Reference daily values were determined for sex and age categories, based on the Dietary Reference Intakes for Japanese, 2020^(29)^, namely, the Recommended Dietary Allowance for protein, vitamins A and C, calcium, iron and magnesium and tentative dietary goal for preventing lifestyle-related diseases for dietary fibre, potassium, saturated fats and sodium. For added sugars, the conditional recommendation advocated by the World Health Organization (i.e. upper limit of 5 % of energy)^(30)^ was used because of the lack of a recommended value for added sugars in Japan, as well as their low intake levels^(31)^. We calculated the NRF9.3 component and total scores based on the daily intake of each nutrient for each participant, which was adjusted for energy intake by the density method and then normalised for the sex- and age-specific Estimated Energy Requirement for a moderate level of physical activity (from the Dietary Reference Intakes for Japanese, 2020^(29)^) and expressed as a percentage of the reference daily value^(19)^. Higher NRF9.3 scores indicated a better quality of the diet. A maximum possible score of 900 indicated a diet in which intakes per given amount of energy were above the reference daily values for the nine qualifying nutrients but below the reference daily values for the three disqualifying nutrients. In the present study, dietary supplements were not considered during the nutrient intake calculation in any of the dietary assessment methods because it was our intention to assess nutrient intake from foods and beverages only.

### Statistical analysis

Statistical analyses were performed using the SAS statistical software (version 9.4; SAS Institute Inc., Cary, NC, USA). A two-tailed *P*-value of <0⋅05 was considered significant. The dietary variables examined in this study included energy-adjusted intakes of sixteen food groups and forty-six nutrients and two diet quality scores (HEI-2015 and NRF9.3). All analyses were conducted for women and men separately. We used the density model for energy adjustment^(32)^. Dietary data were expressed as mean and standard deviation, except for food group intakes for which median and 25th and 75th percentiles were shown.

To assess the estimation ability at the group level, the means and medians of intakes derived from the FCQ were compared with those derived from the DR using paired *t*-test and the Wilcoxon signed-rank test, respectively. The Pearson correlation coefficients (and Spearman correlation coefficient for food groups) between the FCQ and DR estimates were used to assess the ability of the MDHQ to rank individuals in a population. In addition, agreement of the HEI-2015 and NRF9.3 between the FCQ and DR was assessed using the Bland–Altman plot^(33)^. Linear regression analysis was also used to examine the proportional bias between the FCQ and DR^(34)^. Identical analyses were conducted to assess the web FCQ and paper FCQ. As the findings were generally similar, we mainly mention the findings on the web FCQ in the Results section, only briefly describing the findings on the paper FCQ at the very last part.

## Results

Table 1 shows the basic characteristics of 111 women aged 30–69 years and 111 men aged 30–76 years included in this analysis. For both women and men, mean total energy intake derived from the DR was significantly (*P* < 0⋅001) higher than that derived from either the web FCQ or the paper FCQ.

**Table 1.** Basic characteristics of the study population

	Women (*n* 111)		Men (*n* 111)	
	Mean	sd	Mean	sd
Age (years)	49⋅9	10⋅7	51⋅7	11⋅9
Body height (cm)*	158⋅4	5⋅4	170⋅2	6⋅3
Body weight (kg)*	56⋅9	8⋅5	68⋅9	11⋅9
Body mass index (kg/m^2^)^†^	22⋅7	3⋅3	23⋅8	3⋅6
Education level (*n* (%))				
Junior high school or high school	28 (25⋅2)		41 (36⋅9)	
Junior college or technical school	55 (49⋅5)		22 (19⋅8)	
University or higher	28 (25⋅2)		48 (43⋅2)	
Current smoking status (*n* (%))				
Smoker	12 (10⋅8)		35 (31⋅5)	
Non-smoker	99 (89⋅2)		76 (68⋅4)	
Total energy intake (kcal/d)				
4-day DR	1724	335	2286	496
Web version of FCQ	1601	251	1576	321
Paper version of FCQ	1628	213	1565	238

DR, dietary record; FCQ, food combination questionnaire.

* Based on self-report.

† Calculated using the self-reported body height and weight.

### Food group intake

Estimates of energy-adjusted intake of sixteen food groups derived from the DR and web FCQ (and paper FCQ) are summarised in Table 2. The number of food groups (and % of the total number of food groups) for which no statistically significant difference was observed between median intakes estimated using the DR and web FCQ was 6 (38 %) among women and 9 (56 %) among men. The median value of the Spearman correlation coefficients (25th and 75th percentiles) was 0⋅32 (0⋅21–0⋅42) among women and 0⋅38 (0⋅20–0⋅53) among men.

**Table 2.** Median estimates of energy-adjusted intakes of food groups (g/1000 kcal) derived from the 4-day weighed dietary record (DR) and those derived from the web and paper versions of the food combination questionnaire (FCQ), and Spearman correlation coefficients between these estimates in 111 Japanese women and 111 Japanese men*

	Women					Men				
				Spearman Correlation coefficient					Spearman Correlation coefficient	
	DR	Web FCQ	Paper FCQ	DR and web FCQ	DR and paper FCQ	DR	Web FCQ	Paper FCQ	DR and web FCQ	DR and paper FCQ
Rice	128 (75, 155)	169 (135, 201)^c^	169 (139, 201)^c^	0⋅63^c^	0⋅71^c^	141 (98, 182)	182 (139, 219)^c^	181 (146, 218)^c^	0⋅52^c^	0⋅59^c^
Bread	18 (8, 31)	30 (14, 42)^c^	28 (16, 44)^c^	0⋅41^c^	0⋅52^c^	13 (6, 24)	22 (9, 40)^c^	18 (11, 38)^c^	0⋅58^c^	0⋅62^c^
Noodles	36 (12, 59)	43 (33, 60)^b^	40 (32, 60)^b^	0⋅33^c^	0⋅31^b^	43 (18, 73)	46 (34, 60)	43 (30, 56)	0⋅48^c^	0⋅36^c^
Potatoes	16 (8, 25)	16 (14, 19)	16 (14, 19)	0⋅01	0⋅15	12 (7, 20)	16 (13, 19)	16 (13, 20)	0⋅05	0⋅21^a^
Pulses and nuts	26 (10, 48)	25 (21, 28)	24 (20, 29)	0⋅25^b^	0⋅33^c^	19 (10, 30)	23 (19, 26)	23 (19, 28)	0⋅29^b^	0⋅26^b^
Vegetables	114 (83, 156)	128 (93, 148)	137 (104, 157)^a^	0⋅42^c^	0⋅43^c^	102 (74, 135)	105 (76, 151)	130 (96, 161)^c^	0⋅46^c^	0⋅54^c^
Fruit	29 (10, 63)	33 (26, 41)	37 (29, 43)	0⋅42c	0⋅63^c^	13 (0, 33)	29 (23, 36)^c^	30 (22, 38)^c^	0⋅54^c^	0⋅58^c^
Fish and shellfish	25 (16, 36)	27 (24, 29)	27 (24, 29)	0⋅11	0⋅18	24 (15, 37)	27 (23, 30)	27 (25, 31)	0⋅15	0⋅21^a^
Meat	46 (36, 61)	26 (23, 35)^c^	26 (24, 35)^c^	0⋅30^b^	0⋅26^b^	49 (37, 63)	26 (25, 32)^c^	27 (25, 35)^c^	0⋅29^b^	0⋅34^c^
Eggs	19 (12, 29)	16 (15, 17)^c^	17 (16, 18)^b^	0⋅40^c^	0⋅39^c^	18 (12, 26)	17 (14, 19)^a^	17 (16, 19)	0⋅42^c^	0⋅38^c^
Dairy products	60 (23, 120)	55 (34, 80)^a^	61 (41, 90)	0⋅66^c^	0⋅75^c^	28 (6, 82)	37 (18, 63)	47 (19, 67)	0⋅58^c^	0⋅59^c^
Confectioneries	16 (6, 29)	13 (10, 16)^b^	13 (12, 15)^b^	0⋅3^b^	0⋅23^a^	11 (3, 23)	12 (7, 13)	12 (6, 14)	0⋅19	0⋅30^b^
Fruit and vegetable juice	0 (0, 0)	6 (5, 7)^b^	6 (5, 7)^b^	0⋅22^a^	0⋅22^a^	0 (0, 0)	6 (5, 6)^c^	6 (5, 7)^c^	0⋅16	0⋅20^a^
Alcoholic beverages	2 (0, 56)	33 (27, 67)^c^	6 (4, 50)	0⋅64^c^	0⋅59^c^	32 (1, 166)	55 (32, 98)	52 (5, 84)	0⋅62^c^	0⋅70^c^
Soft drinks	0 (0, 38)	11 (8, 17)	13 (8, 18)	0⋅20^a^	0⋅27^b^	0 (0, 48)	11 (7, 17)	11 (8, 17)	0⋅21^a^	0⋅23^a^
Tea and coffee	256 (152, 425)	391 (275, 446)^c^	408 (321, 452)^c^	0⋅20^a^	0⋅21^a^	192 (105, 360)	317 (218, 437)^c^	379 (285, 456)^c^	0⋅35^c^	0⋅38^c^

a *P* < 0⋅05; ^b^*P* < 0⋅01; ^c^*P* < 0⋅001.

* Values are medians (25th and 75th percentiles), unless otherwise indicated. The values derived from the FCQ were compared with those derived from the DR using the Wilcoxon signed-rank test.

### Nutrient intake

Estimates of energy-adjusted intake of forty-six nutrients derived from the DR and web FCQ (and paper FCQ) are summarised in Table 3. The number of nutrients (and % of the total number of nutrients) for which no statistically significant difference was observed between mean intakes estimated using the DR and web FCQ was 17 (37 %) among women and 13 (28 %) among men. The median value of the Pearson correlation coefficients (25th and 75th percentiles) was 0⋅34 (0⋅24–0⋅41) among women and 0⋅31 (0⋅21–0⋅42) among men.

**Table 3.** Mean estimates of energy-adjusted intakes of nutrients (by the density model) derived from the 4-day weighed dietary record (DR) and those derived from the web and paper versions of the food combination questionnaire (FCQ), and Pearson correlation coefficients between these estimates in 111 Japanese women and 111 Japanese men*

	Women					Men				
				Pearson Correlation coefficient					Pearson Correlation coefficient	
	DR	Web FCQ	Paper FCQ	DR and web FCQ	DR and paper FCQ	DR	Web FCQ	Paper FCQ	DR and web FCQ	DR and paper FCQ
Protein (% energy)	15⋅0 ± 2⋅5	13⋅2 ± 0⋅9^c^	13⋅3 ± 0⋅9^c^	0⋅36^c^	0⋅34^c^	14⋅0 ± 2⋅2	12⋅8 ± 0⋅8^c^	13⋅0 ± 0⋅8^c^	0⋅38^c^	0⋅33^c^
Fat (% energy)	31⋅4 ± 4⋅9	26⋅7 ± 2⋅6^c^	26⋅9 ± 2⋅5^c^	0⋅30^b^	0⋅32^c^	28⋅9 ± 5⋅5	25⋅7 ± 2⋅3^c^	26⋅0 ± 2⋅0^c^	0⋅34^c^	0⋅34^c^
SFA (% energy)	9⋅4 ± 2⋅0	7⋅5 ± 1⋅2^c^	7⋅6 ± 1⋅1^c^	0⋅42^c^	0⋅41^c^	8⋅3 ± 1⋅9	7⋅0 ± 1⋅0^c^	7⋅1 ± 0⋅9^c^	0⋅39^c^	0⋅28^b^
MUFA (% energy)	11⋅9 ± 2⋅3	10⋅0 ± 1⋅0^c^	10⋅1 ± 0⋅9^c^	0⋅22^a^	0⋅25^b^	11⋅3 ± 2⋅5	9⋅7 ± 0⋅9^c^	9⋅8 ± 0⋅8^c^	0⋅30^b^	0⋅33^c^
PUFA (% energy)	6⋅4 ± 1⋅5	6⋅1 ± 0⋅4	6⋅2 ± 0⋅4	0⋅23^a^	0⋅24^a^	5⋅9 ± 1⋅5	6⋅1 ± 0⋅4	6⋅1 ± 0⋅3^c^	0⋅26^b^	0⋅45^c^
*n*-6 PUFA (% energy)	4⋅1 ± 1⋅0	4⋅0 ± 0⋅3	4⋅0 ± 0⋅3	0⋅21^a^	0⋅21^a^	3⋅7 ± 1⋅0	3⋅9 ± 0⋅2	3⋅9 ± 0⋅2^a^	0⋅21^a^	0⋅37^c^
*n*-3 PUFA (% energy)	0⋅87 ± 0⋅34	0⋅80 ± 0⋅07^a^	0⋅80 ± 0⋅06^a^	0⋅16	0⋅29^b^	0⋅84 ± 0⋅37	0⋅79 ± 0⋅07	0⋅81 ± 0⋅07	0⋅22^a^	0⋅33^c^
Marine-origin *n*-3 PUFA^†^ (% energy)	0⋅27 ± 0⋅22	0⋅20 ± 0⋅05^c^	0⋅20 ± 0⋅04^c^	0⋅36^c^	0⋅31^c^	0⋅27 ± 0⋅2	0⋅21 ± 0⋅05^b^	0⋅21 ± 0⋅05^b^	0⋅21^a^	0⋅22^a^
EPA (% energy)	0⋅09 ± 0⋅08	0⋅06 ± 0⋅02^b^	0⋅06 ± 0⋅01^b^	0⋅34^c^	0⋅3^b^	0⋅09 ± 0⋅07	0⋅07 ± 0⋅02^b^	0⋅07 ± 0⋅02^a^	0⋅21^a^	0⋅21^a^
*n*-3 DPA (% energy)	0⋅03 ± 0⋅02	0⋅02 ± 0⋅00^c^	0⋅02 ± 0⋅00^c^	0⋅38^c^	0⋅32^c^	0⋅03 ± 0⋅02	0⋅02 ± 0⋅00^c^	0⋅02 ± 0⋅00^c^	0⋅25^b^	0⋅28^b^
DHA (% energy)	0⋅16 ± 0⋅12	0⋅12 ± 0⋅03^c^	0⋅12 ± 0⋅02^c^	0⋅35^c^	0⋅31^b^	0⋅15 ± 0⋅11	0⋅12 ± 0⋅03^b^	0⋅12 ± 0⋅03^b^	0⋅20^a^	0⋅21^a^
α-Linolenic acid (% energy)	0⋅56 ± 0⋅27	0⋅57 ± 0⋅04	0⋅58 ± 0⋅04	−0⋅02	−0⋅03	0⋅54 ± 0⋅30	0⋅56 ± 0⋅04	0⋅57 ± 0⋅03	0⋅08	0⋅25^b^
Cholesterol (mg/1000 kcal)	177 ± 57	138 ± 16^c^	140 ± 16^c^	0⋅29^b^	0⋅33^c^	168 ± 53	137 ± 19^c^	141 ± 18^c^	0⋅31^c^	0⋅27^b^
Carbohydrate (% energy)	50⋅5 ± 6⋅7	56⋅4 ± 3⋅6^c^	56⋅8 ± 3⋅7^c^	0⋅52^c^	0⋅57^c^	49⋅8 ± 7⋅5	56⋅7 ± 3⋅6^c^	56⋅7 ± 3⋅2^c^	0⋅36^c^	0⋅59^c^
Added sugar (% of energy)	6⋅9 ± 3⋅4	4⋅8 ± 1⋅1^c^	4⋅8 ± 0⋅9^c^	0⋅21^a^	0⋅27^b^	6⋅3 ± 4⋅9	4⋅4 ± 1⋅1^c^	4⋅5 ± 1⋅0^c^	0⋅36^c^	0⋅17
Soluble dietary fibre (g/1000 kcal)	1⋅72 ± 0⋅51	1⋅53 ± 0⋅22^c^	1⋅59 ± 0⋅22^b^	0⋅38^c^	0⋅45^c^	1⋅43 ± 0⋅44	1⋅43 ± 0⋅27	1⋅49 ± 0⋅25	0⋅35^c^	0⋅41^c^
Insoluble dietary fibre (g/1000 kcal)	5⋅16 ± 1⋅49	5⋅02 ± 0⋅64	5⋅20 ± 0⋅60	0⋅40^c^	0⋅46^c^	4⋅29 ± 1⋅13	4⋅70 ± 0⋅80^c^	4⋅93 ± 0⋅74^c^	0⋅42^c^	0⋅56^c^
Total dietary fibre (g/1000 kcal)	7⋅1 ± 1⋅9	6⋅9 ± 0⋅9	7⋅1 ± 0⋅9	0⋅41^c^	0⋅48^c^	5⋅9 ± 1⋅5	6⋅4 ± 1⋅2^c^	6⋅8 ± 1⋅1^c^	0⋅43^c^	0⋅55^c^
Alcohol (% energy)	2⋅08 ± 4⋅50	2⋅44 ± 2⋅03	1⋅75 ± 2⋅45	0⋅72^c^	0⋅81^c^	5⋅82 ± 7⋅99	3⋅31 ± 2⋅45^c^	2⋅84 ± 2⋅59^c^	0⋅48^c^	0⋅69^c^
Water (g/1000 kcal)	1307 ± 384	1055 ± 246^c^	1100 ± 270^c^	−0⋅19^a^	−0⋅31^c^	1179 ± 247	748 ± 222^c^	788 ± 198^c^	0⋅18	0⋅16
Retinol (μg/1000 kcal)	110 ± 203	149 ± 31^a^	152 ± 32^a^	−0⋅04	−0⋅03	114 ± 282	143 ± 29	148 ± 25	0⋅10	0⋅06
α-Carotene (μg/1000 kcal)	286 ± 288	240 ± 77	260 ± 69	0⋅28^b^	0⋅25^b^	229 ± 217	222 ± 94	254 ± 88	0⋅24^a^	0⋅26^b^
β-Carotene (μg/1000 kcal)	1335 ± 853	1313 ± 397	1415 ± 358	0⋅34^c^	0⋅32^c^	1074 ± 656	1203 ± 481^a^	1363 ± 458^c^	0⋅31^b^	0⋅32^c^
Cryptoxanthin (μg/1000 kcal)	52⋅0 ± 98⋅4	113⋅5 ± 26⋅0^c^	122⋅0 ± 25⋅6^c^	0⋅17	0⋅25^b^	29⋅6 ± 49⋅0	101⋅5 ± 27⋅5^c^	103⋅9 ± 29⋅4^c^	0⋅18	0⋅28^b^
β-Carotene equivalent^‡^ (μg/1000 kcal)	1524 ± 985	1509 ± 437	1626 ± 395	0⋅35^c^	0⋅32^c^	1223 ± 755	1381 ± 533^a^	1560 ± 507^c^	0⋅29^b^	0⋅32^c^
Retinol equivalent^§^ (μg/1000 kcal)	237 ± 222	276 ± 47	288 ± 50^a^	0⋅03	0⋅08	217 ± 291	259 ± 55	279 ± 50^a^	0⋅13	0⋅08
Vitamin D (μg/1000 kcal)	3⋅45 ± 2⋅30	2⋅72 ± 0⋅47^c^	2⋅73 ± 0⋅40^c^	0⋅35^c^	0⋅39^c^	3⋅09 ± 2⋅00	2⋅70 ± 0⋅50^a^	2⋅81 ± 0⋅50	0⋅12	0⋅41^c^
α-Tocopherol (mg/1000 kcal)	4⋅01 ± 0⋅88	3⋅72 ± 0⋅31^c^	3⋅80 ± 0⋅33^b^	0⋅24^a^	0⋅29^b^	3⋅61 ± 0⋅89	3⋅57 ± 0⋅37	3⋅67 ± 0⋅33	0⋅26^b^	0⋅38^c^
Vitamin K (μg/1000 kcal)	112⋅6 ± 50⋅4	101⋅2 ± 23⋅6^a^	106⋅4 ± 21⋅4	0⋅38^c^	0⋅36^c^	92⋅1 ± 43⋅2	93⋅6 ± 26⋅2	102⋅8 ± 26⋅0^b^	0⋅16	0⋅35^c^
Thiamine (mg/1000 kcal)	0⋅50 ± 0⋅13	0⋅43 ± 0⋅04^c^	0⋅43 ± 0⋅04^c^	0⋅32^c^	0⋅41^c^	0⋅46 ± 0⋅10	0⋅41 ± 0⋅04^c^	0⋅42 ± 0⋅04^c^	0⋅42^c^	0⋅37^c^
Riboflavine (mg/1000 kcal)	0⋅66 ± 0⋅15	0⋅60 ± 0⋅06^c^	0⋅61 ± 0⋅06^c^	0⋅42^c^	0⋅47^c^	0⋅58 ± 0⋅15	0⋅56 ± 0⋅06	0⋅58 ± 0⋅06	0⋅44^c^	0⋅41^c^
Niacine (mg/1000 kcal)	9⋅5 ± 2⋅4	8⋅2 ± 0⋅9^c^	8⋅2 ± 0⋅8^c^	0⋅27^b^	0⋅26^b^	9⋅1 ± 2⋅2	8⋅0 ± 0⋅9^c^	8⋅3 ± 1⋅0^c^	0⋅36^c^	0⋅25^b^
Vitamin B6 (mg/1000 kcal)	0⋅65 ± 0⋅18	0⋅55 ± 0⋅06^c^	0⋅56 ± 0⋅06^c^	0⋅44^c^	0⋅47^c^	0⋅61 ± 0⋅15	0⋅53 ± 0⋅07^c^	0⋅56 ± 0⋅07^c^	0⋅34^c^	0⋅42^c^
Vitamin B12 (μg/1000 kcal)	2⋅83 ± 1⋅61	2⋅92 ± 0⋅46	2⋅93 ± 0⋅42	0⋅28^b^	0⋅33^c^	2⋅62 ± 1⋅50	2⋅90 ± 0⋅48	3⋅00 ± 0⋅48^b^	0⋅16	0⋅20^a^
Folate (μg/1000 kcal)	158 ± 48	164 ± 22	171 ± 21^b^	0⋅26^b^	0⋅36^c^	135 ± 49	152 ± 29^c^	164 ± 27^c^	0⋅24^b^	0⋅36^c^
Pantothenic acid (mg/1000 kcal)	3⋅03 ± 0⋅56	2⋅71 ± 0⋅18^c^	2⋅77 ± 0⋅20^c^	0⋅40^c^	0⋅41^c^	2⋅73 ± 0⋅54	2⋅59 ± 0⋅20^b^	2⋅68 ± 0⋅19	0⋅44^c^	0⋅41^c^
Vitamin C (mg/1000 kcal)	46 ± 19	47 ± 8	50 ± 8^a^	0⋅34^c^	0⋅48^c^	40 ± 17	42 ± 11^c^	46 ± 10^c^	0⋅33^c^	0⋅51^c^
Sodium (mg/1000 kcal)	1937 ± 432	2408 ± 182^c^	2425 ± 155^c^	0⋅19^a^	0⋅19	1900 ± 490	2450 ± 219	2434 ± 187^c^	0⋅34^c^	0⋅2^a^
Potassium (mg/1000 kcal)	1307 ± 299	1200 ± 128	1241 ± 132^b^	0⋅45^c^	0⋅55^c^	1119 ± 246	1115 ± 159^c^	1187 ± 165^c^	0⋅55^c^	0⋅53^c^
Calcium (mg/1000 kcal)	277 ± 96	249 ± 44^c^	258 ± 47^b^	0⋅54^c^	0⋅61^c^	222 ± 85	218 ± 40	231 ± 42	0⋅51^c^	0⋅51^c^
Magnesium (mg/1000 kcal)	141 ± 32	137 ± 11	139 ± 10	0⋅45^c^	0⋅51^c^	123 ± 26	131 ± 13^c^	136 ± 14^c^	0⋅49^c^	0⋅49^c^
Phosphorus (mg/1000 kcal)	555 ± 100	499 ± 34^c^	507 ± 39^c^	0⋅54^c^	0⋅53c	497 ± 93	475 ± 34^b^	489 ± 36	0⋅49^c^	0⋅50^c^
Iron (mg/1000 kcal)	3⋅91 ± 0⋅95	3⋅79 ± 0⋅32	3⋅86 ± 0⋅29	0⋅43^c^	0⋅48^c^	3⋅40 ± 0⋅74	3⋅66 ± 0⋅34^c^	3⋅79 ± 0⋅37^c^	0⋅41^c^	0⋅51^c^
Zinc (mg/1000 kcal)	4⋅40 ± 0⋅77	4⋅01 ± 0⋅23^c^	4⋅06 ± 0⋅23^c^	0⋅24^a^	0⋅25^b^	4⋅06 ± 0⋅66	3⋅93 ± 0⋅26^a^	4⋅02 ± 0⋅23	0⋅47^c^	0⋅38^c^
Copper (mg/1000 kcal)	0⋅58 ± 0⋅12	0⋅57 ± 0⋅04	0⋅58 ± 0⋅04	0⋅42^c^	0⋅46^c^	0⋅52 ± 0⋅09	0⋅57 ± 0⋅04^c^	0⋅58 ± 0⋅04^c^	0⋅45^c^	0⋅51^c^
Manganese (mg/1000 kcal)	1⋅68 ± 0⋅53	1⋅95 ± 0⋅24^c^	2⋅00 ± 0⋅24^c^	0⋅24^a^	0⋅32^c^	1⋅58 ± 0⋅52	1⋅90 ± 0⋅27^c^	2⋅00 ± 0⋅25^c^	0⋅22^a^	0⋅34^c^

SFA, saturated fatty acids; MUFA, monounsaturated fatty acids; PUFA, polyunsaturated fatty acids; EPA, eicosapentaenoic acid; DPA, docosapentaenoic acid; DHA, docosahexaenoic acid.

a *P* < 0⋅05; ^b^*P* < 0⋅01; ^c^*P* < 0⋅001.

* Values are means ± standard deviations, unless otherwise indicated. The values derived from the MDHQ were compared with those derived from the DR using the paired *t*-test.

† Sum of EPA, *n-*3 DPA and DHA.

‡ Sum of β-carotene, α-carotene/2 and cryptoxanthin/2.

§ Sum of retinol, β-carotene/12, α-carotene/24 and cryptoxanthin/24.

### Diet quality score

The total and component scores of HEI-2015 and NRF9.3 derived from the DR and web FCQ (and paper FCQ) are summarised in Table 4. The number of HEI-2015 components (*n* 13 in total) showing no significant mean differences between the DR and web FCQ was five among women and two among men. The number of NRF9.3 components (*n* 12 in total) showing no significant mean differences was six among women and two among men. The Spearman correlation coefficients between the HEI-2015 components derived from the DR and web FCQ ranged from −0⋅14 to 0⋅59 among women and from −0⋅07 to 0⋅54 among men. The corresponding values for the NRF9.3 components ranged from 0⋅06 to 0⋅41 among women and from 0⋅06 to 0⋅51 among men.

**Table 4.** Mean estimates of the total and component scores of Healthy Eating Index-2015 (HEI-2015) and Nutrient-Rich Food Index 9.3 (NRF9.3) derived from the 4-day weighed dietary record (DR) and those derived from the web and paper versions of the food combination questionnaire (FCQ), and Pearson correlation coefficients between these estimates in 111 Japanese women and 111 Japanese men*

	Women					Men				
				Pearson Correlation coefficient					Pearson Correlation coefficient	
	DR	Web FCQ	Paper FCQ	DR and web FCQ	DR and paper FCQ	DR	Web FCQ	Paper FCQ	DR and web FCQ	DR and paper FCQ
HEI-2015^†^	51⋅6 ± 9⋅0	52⋅6 ± 2⋅8	53⋅4 ± 2⋅4^a^	0⋅37^c^	0⋅50^c^	49⋅4 ± 9⋅2	51⋅6 ± 3⋅4^b^	52⋅4 ± 3⋅1^c^	0⋅39^c^	0⋅50^c^
Total fruits	1⋅7 ± 1⋅5	1⋅6 ± 0⋅5	1⋅7 ± 0⋅5	0⋅40^c^	0⋅60^c^	1⋅1 ± 1⋅2	1⋅3 ± 0⋅4^b^	1⋅4 ± 0⋅5^b^	0⋅47^c^	0⋅54^c^
Whole fruits	2⋅6 ± 1⋅9	2⋅9 ± 0⋅9	3⋅2 ± 0⋅9^c^	0⋅40^c^	0⋅62^c^	1⋅7 ± 1⋅8	2⋅5 ± 0⋅9^c^	2⋅6 ± 0⋅9^c^	0⋅49^c^	0⋅55^c^
Total vegetables	4⋅5 ± 0⋅8	4⋅6 ± 0⋅8	4⋅8 ± 0⋅5^c^	0⋅12	0⋅22^a^	4⋅3 ± 1⋅0	4⋅3 ± 1⋅0	4⋅6 ± 0⋅9^b^	0⋅40^c^	0⋅56^c^
Greens and beans	2⋅1 ± 1⋅8	3⋅5 ± 1⋅0^c^	3⋅7 ± 0⋅8^c^	0⋅07	0⋅13	1⋅7 ± 1⋅6	3⋅2 ± 1⋅1^c^	3⋅5 ± 1⋅1^c^	0⋅12	0⋅29^b^
Whole grains	0⋅7 ± 1⋅7	0⋅9 ± 0⋅2	0⋅8 ± 0⋅2	−0⋅14	−0⋅05	0⋅4 ± 1⋅3	1⋅0 ± 0⋅3^c^	0⋅9 ± 0⋅2^c^	−0⋅07	0⋅07
Dairy	2⋅9 ± 2⋅2	2⋅2 ± 1⋅2^c^	2⋅4 ± 1⋅2^b^	0⋅59^c^	0⋅67^c^	1⋅8 ± 1⋅7	1⋅6 ± 0⋅9	1⋅8 ± 1⋅0	0⋅54^c^	0⋅58^c^
Total protein foods	4⋅8 ± 0⋅6	4⋅6 ± 0⋅4^a^	4⋅6 ± 0⋅4^a^	0⋅13	0⋅28^b^	4⋅8 ± 0⋅5	4⋅5 ± 0⋅4^c^	4⋅7 ± 0⋅4^a^	0⋅21^a^	−0⋅04
Seafood and plant proteins	4⋅7 ± 0⋅8	5⋅0 ± 0⋅1^c^	5⋅0 ± 0⋅0^c^	−0⋅04	−0⋅02	4⋅7 ± 0⋅8	5⋅0 ± 0⋅0^c^	5⋅0 ± 0⋅2^c^	NA	0⋅03
Fatty acids^‡^	5⋅9 ± 2⋅8	7⋅4 ± 1⋅7^c^	7⋅3 ± 1⋅6^c^	0⋅42^c^	0⋅42^c^	6⋅5 ± 2⋅4	8⋅2 ± 1⋅4^c^	8⋅0 ± 1⋅5^c^	0⋅31^c^	0⋅33^c^
Refined grains	2⋅3 ± 2⋅8	0⋅2 ± 0⋅8^c^	0⋅2 ± 0⋅8^c^	0⋅49^c^	0⋅53^c^	1⋅9 ± 2⋅7	0⋅1 ± 0⋅5^c^	0⋅1 ± 0⋅5^c^	0⋅34^c^	0⋅52^c^
Sodium	2⋅3 ± 2⋅9	0⋅0 ± 0⋅3^c^	0⋅0 ± 0⋅0^c^	−0⋅01	NA	2⋅6 ± 2⋅9	0⋅0 ± 0⋅0^c^	0⋅0 ± 0⋅0^c^	NA	NA
Added sugars	9⋅2 ± 1⋅2	9⋅97 ± 0⋅18^c^	9⋅99 ± 0⋅06^c^	−0⋅17	−0⋅08	9⋅1 ± 1⋅9	9⋅99 ± 0⋅06^c^	9⋅99 ± 0⋅07^c^	0⋅12	−0⋅03
Saturated fats	8 ± 2⋅1	9⋅7 ± 0⋅7^c^	9⋅6 ± 0⋅8^c^	0⋅34^c^	0⋅30^b^	8⋅8 ± 1⋅5	9⋅9 ± 0⋅3^c^	9⋅9 ± 0⋅4^c^	0⋅27^b^	0⋅16
NRF9.3^§^	588 ± 125	646 ± 53^c^	662 ± 48^c^	0⋅39^c^	0⋅37^c^	586 ± 159	661 ± 65^c^	687 ± 57^c^	0⋅46^c^	0⋅53^c^
Protein	100 ± 1	100 ± 0	100 ± 0	NA	NA	100 ± 1	100 ± 0	100 ± 0	NA	NA
Dietary fibre	76 ± 16	76 ± 10	79 ± 9^a^	0⋅39^c^	0⋅44^c^	73 ± 17	80 ± 14^c^	84 ± 13^c^	0⋅39^c^	0⋅55^c^
Vitamin A	60 ± 21	78 ± 13^c^	81 ± 13^c^	0⋅17	0⋅20^a^	52 ± 22	75 ± 14^c^	81 ± 13^c^	0⋅15	0⋅23^a^
Vitamin C	81 ± 21	89 ± 12^c^	93 ± 10^c^	0⋅27^b^	0⋅28^b^	85 ± 19	93 ± 13^c^	96 ± 11^c^	0⋅33^c^	0⋅43^c^
Vitamin D	67 ± 29	63 ± 10	64 ± 9	0⋅06	0⋅20^a^	74 ± 28	81 ± 12^b^	85 ± 11^c^	0⋅03	0⋅13
Calcium	79 ± 20	75 ± 12	78 ± 13	0⋅48^c^	0⋅61^c^	73 ± 19	76 ± 13	80 ± 13^c^	0⋅45^c^	0⋅49^c^
Iron	85 ± 18	87 ± 14	87 ± 13^a^	0⋅79^c^	0⋅78^c^	97 ± 7	100 ± 0^c^	100 ± 0^c^	0⋅06	0⋅04
Potassium	91 ± 11	91 ± 8	93 ± 7	0⋅28^b^	0⋅31^c^	90 ± 12	93 ± 9^b^	96 ± 8^c^	0⋅51^c^	0⋅48^c^
Magnesium	90 ± 12	93 ± 6^b^	95 ± 5^c^	0⋅27^c^	0⋅33^c^	85 ± 14	92 ± 7^c^	94 ± 7^c^	0⋅41^c^	0⋅41^c^
Added sugars	52 ± 58	8 ± 15^c^	7 ± 11^c^	0⋅21^a^	0⋅23^a^	49 ± 86	5 ± 12^c^	6 ± 12^c^	0⋅40^c^	0⋅12
Saturated fats	38 ± 28	13 ± 14^c^	13 ± 14^c^	0⋅41^c^	0⋅39^c^	26 ± 23	7 ± 9^c^	7 ± 10^c^	0⋅35^c^	0⋅20^a^
Sodium	51 ± 33	87 ± 15^c^	89 ± 13^c^	0⋅26^b^	0⋅25^b^	68 ± 43	116 ± 21^c^	115 ± 19^c^	0⋅36^c^	0⋅25^b^

NA, not available.

a *P* < 0⋅05; ^b^*P* < 0⋅01; ^c^*P* < 0⋅001.

* Values are means ± standard deviations, unless otherwise indicated. The values derived from the FCQ were compared with those derived from the DR using the paired *t*-test. For some variables, the Pearson correlation coefficients were not available because all participants had the same score in the FCQ.

† Calculated as the sum of all components scores. A maximum score is A maximum score for each component is as follows: five for total fruits, whole fruits, total vegetables, greens and beans, total protein foods, and seafood and plant proteins and ten for whole grains, dairy products, fatty acids, refined grains, sodium, added sugars and saturated fats. A higher score indicates a higher diet quality (i.e. a lower intake for refined grains, sodium, added sugars and saturated fats components and a higher intake for other components).

‡ Defined as the ratio of the sum of polyunsaturated fatty acids and monounsaturated fatty acids to saturated fatty acids.

§ Calculated as the sum of scores for nine nutrients to encourage (i.e. protein, dietary fibre, vitamins A, C and D, calcium, iron, potassium and magnesium) minus the sum of scores for three nutrients to limit (i.e. added sugars, saturated fats and sodium). A maximum score is For each component, a maximum score is 100, except for added sugars, saturated fats and sodium components, for which a maximum score is infinite depending on the intake level. A higher score indicates a higher diet quality, except for added sugars, saturated fats and sodium components, for which a higher score indicates an unfavourable dietary intake (i.e. higher intakes of added sugars, saturated fats and sodium).

The mean total scores of HEI-2015 and NRF9.3 derived from the web FCQ were significantly higher than those derived from the DR, except for no difference in the HEI-2015 in women. The Pearson correlation coefficient between the total scores of HEI-2015 derived from the DR and web FCQ was 0⋅37 among women and 0⋅39 among men. The corresponding value for the NRF9.3 total scores was 0⋅39 among women and 0⋅46 among men.

**Fig. 2.**
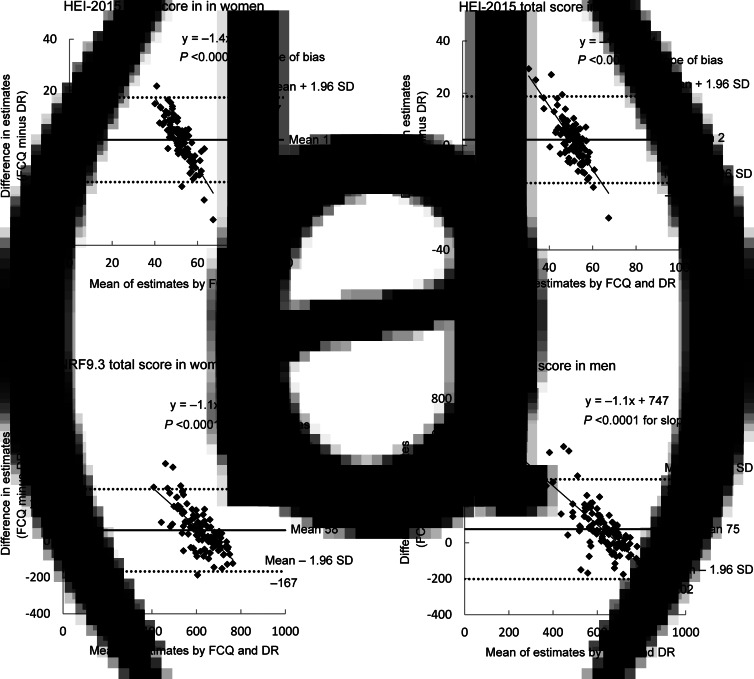
Bland–Altman plots assessing the agreement between estimates of the Healthy Eating Index-2015 (HEI-2015) total score and the Nutrient-Rich Food Index 9.3 (NRF9.3) total score derived from the 4-day weighed dietary record (DR) and those derived from the web version of the food combination questionnaire (FCQ) in 111 Japanese women (a: HEI-2015; c: NRF9.3) and 111 Japanese men (b: HEI-2015; d: NRF9.3). sd, standard deviation.

Fig. 2 shows Bland–Altman plots assessing the agreement between estimates of the HEI-2015 and NRF9.3 total scores derived from the DR and those derived from the web FCQ. The mean difference (FCQ minus DR) was small for the HEI-2015 (+1 among women and +2 among men) but large for the NRF9.3 (+58 among women and +75 among men). In all cases, however, the limits of agreement (mean difference ± 1⋅96 standard deviation of the difference) were wide, indicating poor agreement at the individual level. There was a clear indication of proportional bias between the web FCQ and DR; the HEI-2015 and NRF9.3 total scores tended to be underestimated by the web FCQ as the average score increased.

### The paper version of food combination questionnaire

Identical analyses of the paper FCQ were conducted (Table 2 for food groups, Table 3 for nutrients and Table 4 for diet quality scores; Bland–Altman plots for the HEI-2015 and NRF9.3 total scores not shown). The results for the paper FCQ were generally similar to those for the web FCQ, except for somewhat high Pearson correlation coefficients between the paper FCQ and DR for the total scores of HEI-2015 (0⋅50 among both women and men) and NRF9.3 (0⋅37 among women and 0⋅53 among men).

## Discussion

To our knowledge, this is the first study to examine the relative validity of a newly developed dietary assessment questionnaire specifically designed to capture combinations of food consumed in each meal type (i.e. FCQ) in terms of food and nutrient intakes and overall diet quality against the DR. Overall, the present analysis showed that the FCQ showed an acceptable ability to rank individuals according to consumption for majority of food groups and nutrients as well as diet quality scores. The results on the web and paper versions of the FCQ did not differ substantially. Conversely, the FCQ showed a limited ability to estimate intakes for majority of food groups and nutrients at both the group level and the individual level.

In the present study, only a small proportion of food groups (38–56 %) and nutrients (26–37 %) showed no significant median or mean difference between the FCQ and DR, irrespective of sex and delivery mode. This is generally consistent with the results of previous relative validation analyses of the diet history questionnaire (DHQ) and brief-type diet history questionnaire (BDHQ), the most widely used dietary assessment questionnaires in Japan^(35,36)^. No significant median differences with the 16-day DR were noted for 41–55 % of food groups (varying by sex and dietary assessment questionnaire)^(35)^; moreover, no significant mean differences with the 16-day DR were observed for 10–50 % of nutrients^(36)^. Conversely, for the ranking ability according to food group intake, the FCQ (median correlation coefficients ranging from 0⋅32 to 0⋅38, varying by sex and delivery mode) was not comparable to the DHQ (0⋅43 for women and 0⋅44 for men) or BDHQ (0⋅44 for women and 0⋅48 for men)^(35)^. This was also the case for nutrient intakes; the median correlation coefficients ranged from 0⋅31 to 0⋅35 (depending on sex and delivery mode) in the FCQ, while that ranged from 0⋅47 (men) to 0⋅49 (women) in the DHQ and was 0⋅49 (for both women and men) in the BDHQ^(36)^. The low correlations observed in the FCQ are reasonable given the difference in the number of food items assessed. While only 16 food groups were assessed in the FCQ, the number of food items included in the DHQ and BDHQ was 150 and 58, respectively^(35,36)^. In support of this, a review on dietary assessment questionnaires in Japan showed that long dietary assessment questionnaires (97 or more food items) had slightly higher validity than short counterparts (<70 items) as assessed using correlation coefficients with reference methods^(37)^.

Nonetheless, it should be noted that in the FCQ the correlation coefficients were acceptable (>0⋅30)^(38,39)^ for majority of food groups (56–69 % depending on sex and delivery mode) and nutrients (54–70 %) despite the fact that the FCQ takes much less to complete^(9)^. Furthermore, for the assessment of overall diet quality using the HEI-2015 and NRF9.3, the present study suggests that the FCQ's ability for estimating mean values and for ranking individuals according to diet quality is not inferior to that of the DHQ and BDHQ^(19)^, albeit that the Bland–Altman plots showed poor agreement at the individual level. This is particularly important given that there are few validated dietary assessment questionnaires available for Japanese which can be administered online^(14,40)^ and which assess overall diet quality^(19)^. Because of its comprehensive nature and ease and speed of completion, the FCQ should be considered a strong candidate for a dietary assessment tool in future epidemiologic studies in Japan.

In the present study, the findings for the web FCQ were generally similar to those for the paper FCQ, but Pearson correlation coefficients with the DR were somewhat high for the paper FCQ with regard to overall diet quality measures (HEI-2015 and NRF9.3) compared with the web FCQ. This may be simply because the paper FCQ was completed after conducting the DR, while the web FCQ was completed before conducting the DR. Alternatively, the paper FCQ format, in which questions for each meal are arranged so that they are visible at once, which was not possible with the Web FCQ, may have contributed to a better dietary reporting. While online questionnaires are preferred for administration and processing because they are inexpensive, in real-world settings, not all study participants may be willing to complete the online questionnaires. Thus, further research is needed to investigate potential differences between and the comparability or compatibility of the two modes of FCQ.

Several limitations in the present study warrant mention. First, although the survey was conducted in diverse regions, the present population was not a nationally representative sample of the Japanese population. As volunteers, the participants may have been biased towards greater health consciousness, higher socioeconomic status or both. For example, as mentioned previously^(14)^, the education level in the present population was higher than that in a national representative sample^(41)^. Meanwhile, as mentioned previously^(14)^, the prevalence of current smokers and mean (standard deviation) values of body height, body weight and body mass index in the present participants were similar to those in a nationally representative sample^(42)^. Ideally, further validation should be conducted using a more representative sample.

Second, the weighed DR was used as a reference method; however, the weighed DR is also susceptible to measurement errors due to the erroneous recording and potential changes in eating behaviour^(3)^. However, the weighed DR is the first method of choice for validating the dietary assessment questionnaires because the errors in weighed DR are thought to be less correlated with those in dietary assessment questionnaires compared with the errors in 24-h dietary recall or other instruments that rely on memory^(3,5)^. Additionally, although the dietary recording period was undertaken for 4 d, this duration is unlikely to be sufficient for capturing estimates of habitual intake. Considering that increasing the number of recording days in the reference method improves the apparent validity of a dietary assessment questionnaire^(3,43)^, efforts to increase the duration of recording in the reference method would be important in future validation studies.

Third, the data collection was conducted over a certain period (between August and October 2021; late summer and early autumn in Japan). Considering the seasonal differences in the intake of at least some food groups in Japanese adults^(44–46)^ and that the FCQ only assessed the dietary habits during the previous month, the present data collection would have been conducted throughout the year. However, results of our previous validation study of the DHQ and BDHQ suggested that a single administration of a questionnaire assessing the dietary habits during the previous month may reasonably capture the habitual dietary intake over a longer period (i.e. 1 year)^(19,35,36,47)^. We do not assume that there is any strong reason to consider that the FCQ is an exception in this regard. Finally, our sample size was determined by a widely used recommendation^(3)^ rather than calculation, although our sample size (111 for each sex) is not small compared to previous studies^(3)^.

In summary, compared with the 4-day DR, both the web and paper versions of the FCQ showed acceptable ability for estimating median or mean values and for ranking individuals according to dietary intake for many food groups, nutrients and overall diet quality scores, despite a limited ability to estimate dietary intake at the individual level. Thus, this analysis may lend support to the potential use of the FCQ as a rapid dietary assessment tool in large-scale epidemiologic studies in Japan, although further refinement of this tool should be pursued.
